# INTERPOLAR – prospektive, interventionelle Studien im Rahmen der Medizininformatik-Initiative zur Verbesserung der Arzneimitteltherapiesicherheit in der Krankenversorgung

**DOI:** 10.1007/s00103-024-03890-w

**Published:** 2024-05-15

**Authors:** Markus Loeffler, Renke Maas, Daniel Neumann, André Scherag, INTERPOLAR-Team, F. Meineke, F. Schmidt, S. Stäubert, A. Strübing, M. Yahiaoui-Doktor, M. Nüchter, J. Kaftan, M. Reusche, T. Neumuth, J. Berger, M. Fromm, W. Andrikyan, M. Sponfeldner, F. Dörje, T. Ganslandt, D. Kraska, J. Köck, H. Köster, U. Jaehde, A. Böhmer, T. Bauerdick, S. Zenker, A. Medek, G. Ulrich-Merzenich, M. Coenen, K. Karsten-Dafonte, I. Schulze, M. Kpekpassi, H. Seidling, A. Merzweiler, F. Fritz-Kebede, T. Terstegen, T. Hoppe-Tichy, M. Sedlmayr, I. Reinecke, H. Knoth, A. Fischer, S. Berger, S. Härterich, J. Gewehr, M. Hartmann, K. Farker, M. Kesselmeier, J. Palm, C. Keßler, T. Wendt, S. Franke, V. Koi, F. Richter, C. Sedlaczek, A. Stolz, Y. Remane, K. Strauch, I. Krämer, T. Panholzer, C. Haverkamp, J. Wehrle, M. Hug, D. Tiller, R. Harnisch, A. Dürrbeck, J. Schnurrer, T. Brieden, J. Dedy, A. Michel-Backofen, J. Beck, K. Marquardt, I. Cascorbi, H. Lück, B. Bergh, A. Eisert, D. Wenders, T. Dreischulte, D. Strobach, J. Steinbrech, F. Albashiti, M. Schechner, P. Thürmann, S. Schmiedl, L. Redeker, S. C. Semler, E. Räuscher, K. Green, H. Hilgarth

**Affiliations:** 1 Institut für Medizinische Informatik, Statistik und Epidemiologie (IMISE), Universität Leipzig, Härtelstraße 16–18, 04103 Leipzig, Deutschland; 2 Institut für Experimentelle und Klinische Pharmakologie und Toxikologie, Pharmakologie, Friedrich-Alexander-Universität Erlangen-Nürnberg (FAU), Erlangen, Deutschland; 3 Institut für Medizinische Statistik, Informatik und Datenwissenschaften, Universitätsklinikum Jena, Jena, Deutschland

**Keywords:** Arzneimitteltherapiesicherheit, Medikationsbezogene Probleme, Klinische Entscheidungsunterstützung, Medikationsanalyse, Medikationsmanagement, Drug therapy safety, Medication related problems, Clinical decision support, Medication review, Medication therapy management

## Abstract

Medikationsanalysen durch Stationsapotheker:innen sind eine wichtige Maßnahme der Arzneimitteltherapiesicherheit (AMTS). Dabei werden medikationsbezogene Probleme (Medication Related Problems [MRPs]) identifiziert und zusammen mit den behandelnden Ärzt:innen gelöst. Die Personalressourcen für erweiterte Medikationsanalysen und eine vollständige Dokumentation sind jedoch häufig begrenzt. Bisher müssen Daten, die für die Identifikation von Risikopatient:innen und für eine erweiterte Medikationsanalyse benötigt werden, oft aus verschiedenen Teilen der einrichtungsinternen elektronischen Patientenakte („Electronic Medical Record“ [EMR]) zusammengesucht werden. Dieser fehleranfällige und zeitaufwändige Prozess soll im Projekt INTERPOLAR durch die Nutzung eines durch die Datenintegrationszentren (DIZ) bereitgestellten IT-Werkzeuges verbessert werden.

INTERPOLAR (INTERventional POLypharmacy – Drug InterActions – Risks) ist ein „Use Case“ der Medizininformatik-Initiative (MII), der auf das Thema AMTS fokussiert. Die Planungsphase fand im Jahr 2023 statt, die Routineimplementation ist ab 2024 vorgesehen. AMTS-relevante Daten aus dem EMR sollen dargestellt und die Dokumentation der MRPs in der Routineversorgung erleichtert werden. Die prospektive multizentrische, clusterrandomisierte INTERPOLAR-1-Studie dient dazu, den Nutzen der IT-Unterstützung in der Routineversorgung zu evaluieren. Ziel ist es, zu zeigen, dass mithilfe der IT-Unterstützung mehr MRPs entdeckt und auch gelöst werden können. Dazu werden an 8 Universitätskliniken jeweils 6 Normalstationen ausgewählt, sodass 48 Cluster (mit insgesamt mindestens 70.000 Fällen) zur Randomisierung bereitstehen.

## Abstract (en)

Medication analyses by ward pharmacists are an important measure of drug therapy safety (DTS). Medication-related problems (MRPs) are identified and resolved with the attending clinicians. However, staff resources for extended medication analyses and complete documentation are often limited. Until now, data required for the identification of risk patients and for an extended medication analysis often had to be collected from various parts of the institution’s internal electronic medical record (EMR). This error-prone and time-consuming process is to be improved in the INTERPOLAR (INTERventional POLypharmacy—Drug interActions—Risks) project using an IT tool provided by the data integration centers (DIC).

INTERPOLAR is a use case of the Medical Informatics Initiative (MII) that focuses on the topic of DTS. The planning phase took place in 2023, with routine implementation planned from 2024. DTS-relevant data from the EMR is to be presented and the documentation of MRPs in routine care is to be facilitated. The prospective multicenter, cluster-randomized INTERPOLAR‑1 study serves to evaluate the benefits of IT support in routine care. The aim is to show that more MRPs can be detected and resolved with the help of IT support. For this purpose, six normal wards will be selected at each of eight university hospitals, so that 48 clusters (with a total of at least 70,000 cases) are available for randomization.

## Hintergrund

Unerwünschte Arzneimittelereignisse (Adverse Drug Event [ADE]) tragen erheblich zur Morbidität und Mortalität im Krankenhaus bei [1–4]. In der Routine werden medikationsbezogene Probleme (Medication Related Problems [MRPs]) häufig nicht oder zu spät als solche erkannt, was zu einer Verschlimmerung und/oder Verstetigung führen kann [5, 6]. ADE sind auch ein relevanter Kostenfaktor, mit einer konservativen Schätzung von vermeidbaren 1,3 Mrd. € allein in deutschen Notaufnahmen [7]. Ein großer Teil dieser MRPs, insbesondere solche, die durch Interaktionen oder Medikationsfehler entstehen, gelten als vermeidbar. Eine Mitbetreuung von Patient:innen durch Stationsapotheker:innen kann MRPs verringern, was in der klinischen Routine jedoch oft schwer zu belegen ist [8–10].

Eine der Kernaufgaben von Stationsapotheker:innen ist die Medikationsanalyse bei stationären Patient:innen zur Identifikation von MRPs. Umfang und Tiefe der Medikationsanalyse reichen von einfachen Prüfungen auf Wechselwirkungen auf Basis des Medikationsplans bis hin zu umfassenden Analysen unter zusätzlicher Berücksichtigung von Diagnosen, Laborwerten und Patientengesprächen. Sie richten sich u. a. nach den verfügbaren Ressourcen und es hängt von ihnen ab, welcher Anteil an Medikationsproblemen identifiziert werden kann und welche Fehlalarme vermieden werden können [11]. Je nach Anzahl der Medikamente und relevanter Komorbiditäten dauert eine „erweiterte“ Medikationsanalyse, also eine standardmäßige pharmazeutische/pharmakologische Kurvenüberprüfung im eigentlichen Sinn, einschließlich der Dokumentation von gefundenen Problemen und Empfehlungen und Kommunikation mit Behandelnden/Pflegenden oder Patient:innen, zwischen 15 min und 60 min [12–15]. Es ist auch zu beachten, dass erweiterte oder umfassende Medikationsanalysen, bei denen nicht nur die Medikamente, sondern auch Diagnosen und das Labor berücksichtigt werden – und ggf. Patient:innen direkt befragt werden –, zeitaufwändig sind. Ihre Effektivität hängt dabei auch maßgeblich von der Akzeptanz der vorgeschlagenen Handlungsempfehlungen durch die verordnenden Ärzt:innen ab. Im Jahr 2021 beschäftigten von 1887 deutschen Krankenhäusern nur 356 (18,9 %) Stationsapotheker:innen, mit im Mittel 5,8 Vollzeitäquivalenten. Dadurch wird deutlich, dass der Idealzustand von täglichen erweiterten Medikationsanalysen bei allen Patient:innen mangels Fachkräfte nicht realisierbar ist [16].

In der Routineversorgung identifizieren die Stationsapotheker:innen solche Patient:innen, die für eine erweiterte oder umfassende Medikationsanalyse und/oder eine Patientenvisite infrage kommen. Dieses Vorscreening der elektronischen Patientenakten ist nicht strukturiert und daher ineffizient. International sind zur Wahrung der Arzneimitteltherapiesicherheit (AMTS) mehrere Scores zur Identifikation von Risikopatient:innen publiziert worden. Deren technische und direkte Implementation in einem deutschen Krankenhaus steht aber bisher entgegen, dass sie vielfach in anderen Gesundheitssystemen und für spezifische Settings entwickelt wurden. Erschwerend kommt hinzu, dass sie für eine digitale Umsetzung zu komplex sind und zu viele Datenquellen erfordern, die in Deutschland oft nicht rechtzeitig digital codiert verfügbar sind [17]. Zur Unterstützung von Ärzt:innen und Stationsapotheker:innen bei der Erkennung von MRPs wurden zahlreiche Kriterienlisten entwickelt [18–20]. Für eine direkte Ressourcensteuerung durch Krankenhausapotheker:innen im Sinne einer Risikodifferenzierung zwischen Patient:innen sind diese Risikolisten für sich allein aber noch nicht gut geeignet, da sie in der klinischen Routine schlicht zu viele „Treffer“ liefern und nicht auf die lokale klinische Relevanz abgestimmt sind. In einer aktuellen Analyse von 154.048 stationär behandelten geriatrischen Patient:innen fand sich bei 77 % mindestens ein Wirkstoff der PRISCUS‑2.0‑Liste, auf der potenziell ungeeignete Arzneimittel für ältere Menschen verzeichnet sind [21].

Die mangelnde Verfügbarkeit arzneimitteltherapierelevanter Daten für die Bewertung der Medikationssicherheit schränkt die Möglichkeiten der in Deutschland bereits eingesetzten klinischen Entscheidungsunterstützungssysteme „Clinical Decision Support Systems – CDSS“ oft ein [22]. Kommerziell verfügbare elektronische Verordnungsinstrumente mit CDSS zur Erkennung von MRPs werden in der Mehrzahl der deutschen Universitätskliniken noch nicht in einem für eine erweiterte Medikationsanalyse ausreichenden Funktionsumfang eingesetzt, der auch Diagnosen und Laborwerte berücksichtigt. Klinisch einheitliche Validierungen der Systeme (und der Prozesse, in denen sie eingesetzt werden) im Routinebetrieb sind uns nicht bekannt [23].

Insgesamt werden die notwendige Tiefe und Vollständigkeit der Dokumentation von MRPs (insbesondere auch, ob und wie sie gelöst wurden oder ob und wieso diese fortbestehen) in der klinischen Routine oft nicht erreicht. Viele MRPs können in der Routine auch nicht passend codiert werden. In einigen deutschen Krankenhäusern werden MRPs und die dazugehörigen pharmazeutischen Interventionen in der Datenbank des Bundesverbandes deutscher Krankenhausapotheker (ADKA) DokuPik anonymisiert dokumentiert. ADKA DokuPik ist nicht für die innerklinische fallbezogene (personenbeziehbare) Dokumentation der pharmazeutischen Tätigkeit oder Kommunikation zwischen Behandlern ausgelegt, sondern dient nur als summarische anonyme Datenerfassung für Forschung und übergreifende Statistiken.

Im Rahmen des INTERPOLAR^1^-Vorhabens, eines neuen „Use Case“ der Medizininformatik-Initiative (MII) seit 2023, soll eine fallbezogene lokale Dokumentation der MRPs durchgeführt werden. Die Routineimplementation ist ab 2024 vorgesehen. Dieser Artikel beschreibt die Rationale, das Design, die technische Umsetzung und die Evaluationsmethodik von INTERPOLAR.

## Vorerfahrungen aus dem Projekt „POLAR_MI“ und Ziele des INTERPOLAR-Vorhabens

In dem vorangegangenen übergreifenden Use Case „POLAR_MI“ der MII haben die Teilnehmenden gelernt, wie Datenausleitungen zum Thema AMTS aus vielen Standorten machbar sind und welche Herausforderungen hierbei auftreten [24]. Es war möglich, die Daten von 790.000 stationären Fällen aus 9 Kliniken zwischen 2018 und 2021 auszuleiten. Die Häufigkeiten bestimmter Verordnungen konnten auf der Basis verschiedener Profile des MII-Kerndatensatzes ermittelt werden. Damit war es möglich, die Verordnung von potenziell inadäquaten Medikationen (PIM) bei Patient:innen über 65 Jahren zu zählen. Wir fanden, dass es je nach Referenz (PRISCUS 1.0, PRISCUS 2.0, EU(7)-PIM^2^, s. oben) Verordnungen von PIM-Wirkstoffen bei 14 %, 40 % oder 47 % der Patient:innen gab. Die wichtige Botschaft des POLAR-Projektes war, dass die technischen und organisatorischen Schwierigkeiten bei der Ausleitung und Analyse von AMTS-Daten wahrscheinlich beherrschbar sind und damit eine ambitioniertere Vorgehensweise mit prospektiver Datenausleitung wie in INTERPOLAR in Reichweite gelangt.

Die Studie „INTERPOLAR-1“ als Teil des Projektes INTERPOLAR verfolgt das Ziel, die Arbeitsprozesse der Stationsapotheker:innen in der Routineversorgung mit IT-Unterstützung effizienter zu gestalten. Es sollen Risikopatient:innen, die besonders von den erweiterten Medikationsanalysen profitieren, schneller und zuverlässiger identifiziert werden. Folglich sollen vorhandene medikationsbezogene Probleme bei einzelnen Patient:innen schneller erfasst und gelöst werden.

Neben einer verbesserten Nutzung der Daten aus der elektronischen Patientenakte („Electronic Medical Record“ [EMR]) der Krankenhausinformationssysteme (KIS), die über die lokalen Datenintegrationszentren (DIZ) integriert werden, soll in INTERPOLAR‑1 insbesondere die Dokumentation der identifizierten MRPs verbessert werden.

Um die an der Studie teilnehmenden Stationsapotheker:innen informationstechnisch zu unterstützen, soll ein „AMTS-Cockpit“ eingesetzt werden, das auf einer grafischen Benutzeroberfläche gezielte Übersichten zu den Daten der Patient:innen bereitstellt und folgende Funktionen aufweist:

1. Erleichterte Erkennung von Patient:innen mit erhöhtem Risiko für MRPs durch:
  - frei sortierbare Darstellung von Faktoren, die üblicherweise zur Beurteilung des individuellen Risikos für Medikationsprobleme benötigt werden (AMTS-relevante Diagnosen, Laborwerte etc.),
  - frei sortierbare Darstellung von Verdachtssignalen (sog. Trigger) für bereits vorliegende Medikationsprobleme,
2. Erleichterte Dokumentation der Medikationsanalysen:
  - Erfassung der Patient:innen, bei denen Medikationsanalysen durchgeführt wurden,
  - Erfassung aufgefundener Medikationsprobleme und der verfolgten Lösung (z. B. Kontaktaufnahme mit den behandelnden Ärzt:innen).

Im weiteren Verlauf von INTERPOLAR ist zur externen Validierung der Ergebnisse aus INTERPOLAR‑1 eine weitere Beobachtungsstudie (INTERPOLAR-2) vorgesehen.

## Die Studie INTERPOLAR-1

### Studiendesign.

INTERPOLAR‑1 ist eine multizentrische „stepped-wedge cluster-randomisierte“ (SW-CRT) Studie. Hierbei sind die Stationen und nicht die Patient:innen die Einheit der Randomisierung. Das Design hat den Vorteil, dass es logistisch besser in den Routineprozess integrierbar ist, da nicht Patient:innen individuell randomisiert werden, sondern größere Cluster – in unserem Fall Stationen. Die Medikationsanalyse durch die Stationsapotheker:innen ist ebenfalls stationsbezogen.

**Abb. 1:**
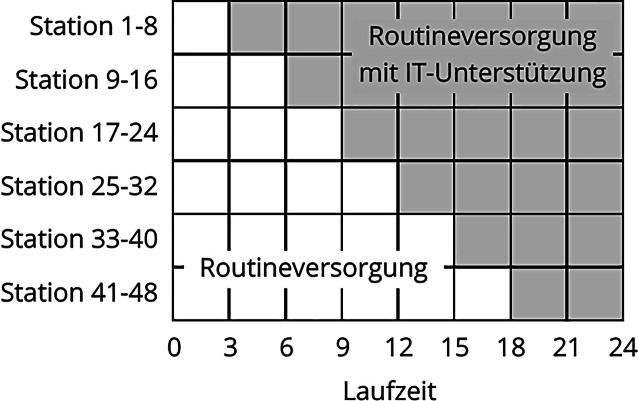
Studiendesign und Ablaufplan der Studie INTERPOLAR‑1, wobei zufällig Stationen der teilnehmenden Kliniken zu einzelnen Clustern zusammengefasst werden. (Quelle: eigene Abbildung)

Die Studie INTERPOLAR‑1 wird in 2 Phasen unterteilt (Abb. 1):

- *Phase 1* – „Usual Care“ (UC): Routineversorgung von Patient:innen durch Stationsapotheker:innen,
- *Phase 2* – „Usual Care“ mit IT-Unterstützung (UC + IT): Routineversorgung von Patient:innen durch Stationsapotheker:innen. Die Stationsapotheker:innen erhalten das „AMTS-Cockpit“, das Informationen zur AMTS-Risikobewertung der Patient:innen aus den in den KIS verfügbaren EMRs darstellt.

### Studienpopulation.

Aufgrund des clusterrandomisierten Designs und der Einbeziehung der DIZ in die Analyse gibt es 2 Ebenen des Ein- und Ausschlusses, die Stationsebene und die Patientenebene. Bei den ausgewählten Stationen handelt es sich um Stationen, deren Patient:innen in der Routine bereits von Stationsapotheker:innen versorgt werden und bei denen mindestens 2 × pro Woche eine Medikationsanalyse erfolgt. Die medizinischen Behandlungsdaten lassen sich über die DIZ in die INTERPOLAR-Datenbank ausleiten. Es gibt keine patientenbezogenen Ein- bzw. Ausschlusskriterien. Alle Patient:innen in der Versorgung auf den INTERPOLAR-Stationen werden eingeschlossen. Lediglich bei der Auswertung werden Patient:innen unter 18 Jahren weggelassen. Es gibt keine patientenbezogenen oder fallbezogenen Rekrutierungsmaßnahmen.

**Abb. 2:**
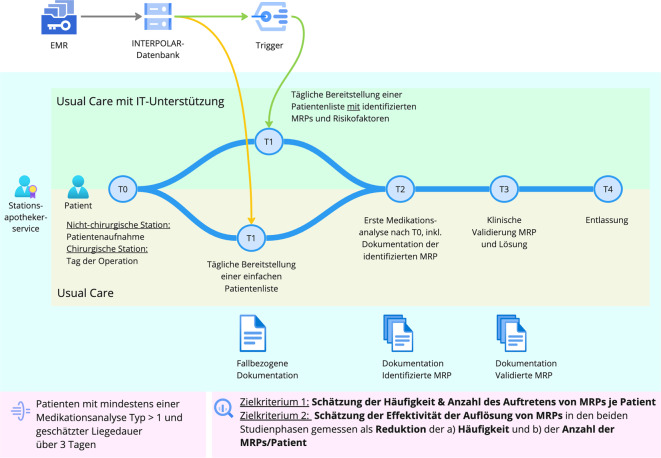
Ablauf der Intervention der Routineversorgung in den 2 Phasen (UC und UC + IT) – mit IT-Unterstützung (*grün*) des Stationsapothekerdienstes und „as usual“ (*gelb*). (Quelle: eigene Abbildung)

Die INTERPOLAR-1-Studie (Abb. 2) ist in 2 Phasen unterteilt: 1.) Usual Care (UC; gelb) und 2.) Usual Care mit IT-Unterstützung (UC + IT; grün). Einziger Unterschied zwischen den Studienphasen ist, dass die Stationsapotheker:innen in der 2. Studienphase auf die volle IT-Unterstützung des AMTS-Cockpits zurückgreifen können. Der Stationsapothekerservice auf den beteiligten Stationen bleibt unverändert. Der Zeitpunkt des Wechsels von der ersten in die 2. Phase, also die Umstellung auf die IT-unterstützte Routineversorgung, erfolgt für jede Station entsprechend der Clusterrandomisierung.

### Studienziele.

Das primäre Ziel von INTERPOLAR‑1 ist es, in einem multizentrischen Setting den konfirmatorischen Nachweis zu erbringen, dass bei Usual Care *mit* operationalisierter IT-Unterstützung (UC + IT) insgesamt mehr MRPs erkannt und behoben werden als bei Usual Care (UC).

Primärer Endpunkt ist ein MRP. Ein MRP liegt vor, wenn eine schwerwiegende, als klinisch relevant beurteilte Kontraindikation oder ein vergleichbar schwerwiegendes Problem vorliegt (z. B. Dosierungsproblem, Anwendungsproblem, nicht gegebene dringend indizierte Medikation).

Folgende Zielkriterien wurden festgelegt:

*Zielkriterium 1:* Eine Schätzung der Anzahl des Auftretens von identifizierten und dokumentierten MRPs/Fall, getrennt nach den Studienphasen (UC vs. UC + IT). Das Zielkriterium 1 wird für alle Fälle ermittelt, für die eine MRP-Bestimmung durch Stationsapotheker:innen möglich war.

*Zielkriterium 2:* Die Schätzung der Zahl der gelösten MRPs pro Fall, getrennt in den beiden Studienphasen. Das Zielkriterium 2 wird für alle Fälle ermittelt, bei denen eine Lösung der initial aufgefundenen MRPs ermittelbar war.

Des Weiteren sollen folgende sekundäre Auswertungsziele verfolgt werden:

- Schätzung der Kohortenprävalenz von MRPs,
- Analyse der Profile der aufgetretenen MRPs,
- Analyse der Profile der behobenen MRPs,
- Vorhersagbarkeit von MRPs (Regressionsmodelle),
- Identifikation von „Adverse Drug Events“ (ADE) mittels algorithmisierbarer Klassifikatoren,
- Aufwand der Erfassung und Lösung von MRPs in der Routineversorgung.

### Datenerfassung und Datenpräsentation.

Im Grundsatz werden alle fallbezogenen Daten und die zugehörigen vorliegenden klinischen Informationen (Aufnahmediagnosen, Medikationen, Laborwerte, Risikofaktoren) aus der EMR extrahiert und in eine lokale Datenbank (INTERPOLAR-DB) geladen. Die Stationsapotheker:innen werden dort die von ihnen festgestellten MRPs, ihre Handlungsempfehlungen und Lösungsvorschläge zu den MRPs (für ihre interne Verwendung und primär für die Organisation ihrer Tätigkeit und ihre eigene Qualitätssicherung) dokumentieren. Diese apothekeninterne Tätigkeitsdokumentation und Qualitätssicherung werden an den Standorten harmonisiert und digital unterstützt, sie erfolgten bisher unsystematisch. Die Apothekendokumentation ist nicht zu verwechseln mit der Mitteilung etwaiger bei der Medikationsanalyse gefundener MRPs an die behandelnden Ärzt:innen, die je nach Dringlichkeit in verschiedenen Modalitäten, wie Telefon oder Aktennotiz, erfolgt. Den behandelnden Ärzt:innen obliegt die finale Entscheidung über eigentliche Therapieanpassung und ggf. Dokumentation in der EMR. Es ist wichtig hervorzuheben, dass sich hier nichts an der bisherigen Dokumentationsroutine ändert.

In der UC + IT-Phase des Vorhabens kommen *operationalisierte Listen von Kontraindikationen* zum Einsatz, die im INTERPOLAR-Konsortium ausgearbeitet wurden. Diese umfassen:

- Wirkstoff/Wirkstoff-paarweise Kontraindikationen (ca. 540 Paare sind gelistet mit ATC-Codes^3^),
- Wirkstoff/Wirkstoffgruppen-Kontraindikationen (ca. 250 Arzneimittel mit größeren Gruppen von je 10–30 Arzneimitteln sind gelistet, z. B. MAO-Hemmer, Tetrazykline),
- Wirkstoff/Diagnose-Kontraindikationen (ca. 200 Arzneimittel für bestimmte Erkrankungen mit ICD-10-Codes gelistet, Erkrankungen werden zudem im Expertenkonsensus sowohl aus Laborwerten als auch verordneten Medikamenten ermittelt),
- Wirkstoff/Laborwert-Kontraindikationen (ca. 100 Arzneimittel gelistet, insbesondere eingeschränkte Nierenfunktion und andere Laborparameter mit LOINC-Codes^4^ und hierzu im Expertenkonsensus ermittelte Schwellenwerte).

Diese Listen stellen den Kern der UC + IT-Vorgehensweise dar.

In der UC + IT-Phase wird bei jeder Datenausleitung von EMR-Daten eines Falles geprüft, ob eines oder mehrere der Kontraindikationskriterien erfüllt sind. Es wird dann aus dem Algorithmus heraus jeweils ein Trigger „MRP-Verdacht“ gesetzt. Dieser Trigger wird in das AMTS-Cockpit eingespielt und den Stationsapotheker:innen zur Kenntnis gebracht. Sie können nun die Detailprüfung vornehmen und entscheiden, ob sie ein tatsächliches MRP feststellen und eine Handlungsempfehlung umgesetzt wird.

Wir gehen davon aus, dass durch diese IT-gestützte operationalisierte Suche nach möglichen MRPs eine erhebliche Zahl von weniger häufigen bis seltenen, aber klinisch relevanten MRPs aufgefunden wird, die bei einer Suche unter UC eher übersehen werden. Eine Befragung der INTERPOLAR-Beteiligten (klinischen) Pharmakolog:innen und Stationsapotheker:innen (*n* = 19) hat ergeben, dass man durch das UC + IT-Verfahren eine Steigerung der entdeckten relevanten MRPs um 10–25 % erwartet. Zudem wird erwartet, dass sich von diesen nochmals 10–20 % mehr lösen lassen als bei den unter UC entdeckten MRPs. Eine solche Größenordnung wäre eine deutliche Verbesserung der AMTS. Das ist die Rationale für das INTERPOLAR-Vorhaben.

Die Routinedokumentation der MRPs durch die Stationsapotheker:innen umfasst:

- Datum der Medikationsanalyse und deren Tiefe,
- Feststellung, ob tatsächlich ein MRP vorliegt,
- Feststellung, welcher Art das MRP ist (z. B. Art von Kontraindikation, übersehene Indikation),
- Handlungsempfehlung,
- Überprüfung, ob eine Lösung des MRP im Verlauf erfolgt ist,
- Abschluss der MRP-Prüfung.

### Auswertungskollektive.

Für INTERPOLAR‑1 erfolgt eine geschachtelte Definition der Auswertungskollektive, hier „Full Analysis Set“ (FAS) 1–3 genannt, die sich allerdings erst zum Zeitpunkt der Datenauswertung ergibt.

*Grundgesamtheit (FAS1):* alle Patient:innen, die während der Laufzeit der Studie für mindestens 1 Tag (d. h. über Nacht) auf einer der eingeschlossenen Stationen waren und bei Aufnahme mindestens 18 Jahre alt waren.

*Prävalenzpopulation (FAS2):* Das sind zunächst alle Fälle (Untergruppe von FAS1), bei denen eine MRP-Identifikation möglich war (bei chirurgischen Patient:innen sollte die MRP-Identifikation erst frühestens 2 Tage postoperativ beginnen). Hier wird die Art der MRPs und der Aktion ausgewertet. Das erlaubt eine approximative Prävalenzschätzung der MRPs an deutschen Universitätskliniken.

*Lösungspopulation (FAS3):* Für die Bestimmung des primären Zielkriteriums bzgl. der Lösbarkeit von MRPs werden nur die Fälle herangezogen, bei denen ein MRP dokumentiert wurde und bei dem die MRP-Lösung nach MRP-Identifikation erfolgen konnte. Die Lösungspopulation ist eine strikte Teilmenge der Prävalenzpopulation (FAS2).

*Erwartete Rekrutierung: *Wir rechnen je nach ausgewählten Stationen und nach der Dauer der INTERPOLAR-1-Studie mit 75.000–103.000 Fällen in der FAS1-Population. Wir erwarten überschlägig, dass für die FAS2-Population rund ein Viertel weniger Fälle zur Verfügung stehen wird (Kurzlieger, MRP-Beurteilung nicht schnell genug möglich, nicht informative Daten). Die FAS3-Population wird nochmals kleiner sein als die FAS2-Population. Sie wird davon bestimmt, ob es möglich ist, die MRP-Lösung zu ermitteln. Das setzt voraus, dass die Patientin oder der Patient noch stationär ist und die Daten im EMR vorliegen. Wir rechnen mit einem Informationsverlust von weiteren 20–25 %. In den Powerkalkulationen für das Zielkriterium 2 rechnen wir daher mit 42.000 informativen Fällen in FAS3.

### Fallzahlplanung.

Die Fallzahlberechnung findet in Anlehnung an den von Hussey und Hughes [25] vorgeschlagenen Ansatz statt. Eingesetzt wird die Statistiksoftware R mit dem Paket „swGlmPwr“, welches die statistische Power des spezifizierten SW-CRT-Designs im Kontext verallgemeinerter linearer Modelle wiedergibt. Die statistische Power wurden in Abhängigkeit der folgenden Parameter evaluiert:

- Anzahl der Stationen pro Randomisierungsschritt,
- durchschnittliche Anzahl an Fällen pro Station und Zahl der Wochen,
- Variabilität zwischen den Clustern bzgl. des Auftretens von MRPs,
- Anzahl der MRPs unter UC,
- Anteil gelöster MRPs unter UC und unter UC + IT.

Wir kommen zu dem Schluss, dass wir bei 40.000 Fällen genug Power (> 80 %) haben, um im konservativsten Fall einen Zugewinn in der MRP-Anzahl (Prävalenz) von mindestens 5 Prozentpunkten zu erreichen und proportional analog mehr gelöste MRPs zu identifizieren. Ein mögliches Szenario ist in Tab. 1 aufgezeigt.

**Tab. 1:** Szenario eines möglichen Studienergebnisses in Hinblick auf den Zugewinn an identifizierten medikationsbezogenen Problemen (Medical Related Problems [MRPs])

Zielkriterien	Usual Care (UC)	Usual Care und IT-Unterstützung (UC + IT)	Zugewinn
*Zielkriterium 1*: gefundene MRPs pro 1000 Fälle (Prävalenz)	100/1000 (10 %)	150/1000 (15 %)	50 MRPs mehr entdeckt (5 Prozentpunkte)
*Zielkriterium 2*: gelöste MRPs mit Lösungsrate von 50 % in beiden Studienphasen	50/100	75/150	25 MRPs mehr gelöst (Zugewinn um 50 %)

### Datenfluss und Datenmanagement.

**Abb. 3:**
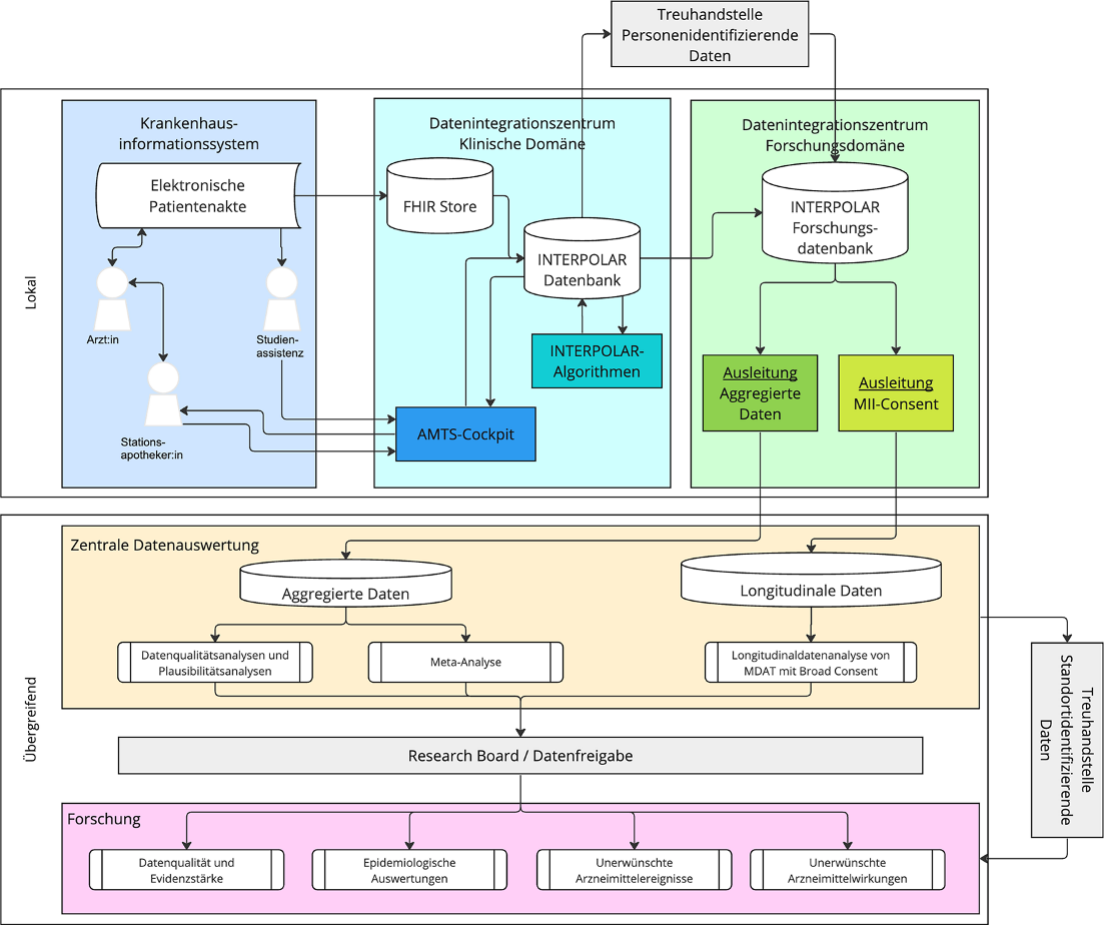
IT-Architektur und Datenfluss der INTERPOLAR-1-Studie von der Routineversorgung zu den Forscher:innen. (Quelle: eigene Abbildung)

Die Falldaten werden an den Universitätskliniken durch ihre DIZ aus den KIS (EMR) extrahiert und in interoperable Formate gemäß MII-Kerndatensatzstruktur (HL7 FHIR^5^) überführt. Der Datenfluss ist in Abb. 3 dargestellt. Für die Erschließung der Falldaten aus den klinischen Anwendungssystemen liegt an den 8 beteiligten Standorten ein Datenschutzkonzept vor.

Es sind lokal folgende Software-Komponenten aufzubauen:

1. *ETL-Prozess zur Abfrage der für INTERPOLAR relevanten Daten aus der EMR: *Das impliziert eine genaue Festlegung aller FHIR-Ressourcen, die zum Einsatz kommen müssen („Catalog of Items“, CoI). Hier greifen wir auf Erfahrungen im Vorgängerprojekt POLAR_MI zurück. Wir benötigen z. B. FHIR-Ressourcen wie Observations, Medication Statement, Medication Request. Wesentlich sind hier die Datenausleitungen zu den operationalisierten Kontraindikationslisten. Die Datenausleitungen werden mit dem vom DIZ betriebenen FHIR-Server mindestens einmal täglich nachts erfolgen.
2. *Die INTERPOLAR-Datenbank:* Die DIZ sollen jeweils eine lokale INTERPOLAR-DB betreiben. Merkmalsfelder werden durch einen „Catalog of Items“ beschrieben, der verbindlich ist. Darin werden einerseits die über die FHIR-Ressourcen eingehenden Daten strukturiert abgelegt und andererseits auch die durch das Dokumentationswerkzeug entstehenden Eintragungen durch die Stationsapotheker:innen verwaltet. Diese INTERPOLAR-DB enthält die Patientendaten, der an INTERPOLAR teilnehmenden Stationen, die durch die Stationsapotheker:innen dokumentierten MRPs (UC) sowie die durch die INTERPOLAR-Algorithmen berechneten MRPs und deren Bewertung durch die Stationsapotheker:innen (UC + IT).
3. *Das AMTS-Cockpit* hat 2 Funktionen. Es bietet den Zugang zur INTERPOLAR-Datenbank sowie den dort verfügbaren Patientendaten (fallbasiert) und Möglichkeiten für die Stationsapotheker:innen, in diesen Daten zu navigieren. Das AMTS-Cockpit ermöglicht die MRP-Dokumentation durch die Stationsapotheker:innen.

### AMTS-Cockpit.

Im Projekt ist vorgesehen, dass Stationsapotheker:innen auf Basis angezeigter Informationen schneller Patient:innen mit hohem Risiko für MRP identifizieren können und bei diesen gezielt eine Medikationsanalyse durchführen. Die Medikationsanalyse soll durch die aus der EMR zusammengefassten AMTS-relevanten Daten und ggf. bereits identifizierte potenzielle MRPs beschleunigt werden. Der Studienzweck besteht darin, zu zeigen, dass eine solche Prozessoptimierung in der Versorgung hilft, mehr relevante MRPs zu detektieren und diese aufzulösen. Folglich soll so die Arzneimitteltherapiesicherheit verbessert werden. Hierfür soll ein spezielles IT-gestütztes Verfahren die vorliegenden Behandlungsinformationen und Falldaten den Stationsapotheker:innen für die Medikationsanalyse vorlegen (UC + IT). Die Aufbereitung von relevanten Informationen aus den vorliegenden EMR-Daten erfolgt durch einen Abgleich mit hinterlegten Listen. Der Abgleich produziert auf Grundlage von vordefinierten Regelwerken die sogenannten Trigger. Die Vorlage relevanter Informationen und Trigger (UC + IT) als auch die Dokumentation von MRP (UC und UC + IT) erfolgen im AMTS-Cockpit. Wir nehmen an, dass das AMTS-Cockpit – auch wenn im Wesentlichen nur Daten aus anderen Systemen angezeigt werden – für den vollen Funktionsumfang (auch Anzeige von Kontraindikationsrisiken) als Medizinprodukt nach Medical Device Regulation (MDR) einzustufen ist.

Zur Durchführung der Studie werden einzelne Komponenten einer Referenzarchitektur sowie die Studienanforderungen erarbeitet und zur Verfügung gestellt. Für die regulatorische Einordnung nach MDR ist es wichtig zu verstehen, dass Software-Komponenten und Daten nicht einfach lokal installiert und betrieben werden können, sondern lokal – z. T. erheblich – angepasst werden müssen. Dies mündet in einer Reihe technischer Lösungen – stark abgestimmt auf lokale Besonderheiten. Im Augenblick wird deshalb geklärt, inwieweit dieses Vorgehen an jedem Standort eine „Eigenherstellung“ im Sinne des MDR darstellt [26]. Die Standorte werden im Rahmen des Projekts dann bei der Erstellung und Implementation der hier erforderlichen Qualitätssicherungsmaßnahmen anhand von „Best-Practice“-Beispielen im Sinne der gegenseitigen Hilfe zur Selbsthilfe aus dem Konsortium unterstützt.

Die Auswertung erfolgt nach dem Prinzip der verteilten Datenanalyse nach MII-Standard. Dazu werden die Daten aus der INTERPOLAR-Datenbank unter Verwendung der lokalen Treuhandstelle pseudonymisiert. Anschließend werden Daten-Aggregate ohne Personenbezug (z. B. Schätzer relativer Häufigkeiten) gebildet und für die zentrale Analyse bereitgestellt.

## Diskussion

INTERPOLAR ist ein „Use Case“ der Medizininformatik-Initiative (MII), der auf das Thema AMTS fokussiert. Hier haben wir Rationale und das Design einer großen interventionellen Studie (INTERPOLAR-1) vorgestellt, die im Jahr 2024 starten wird. Wir haben basierend auf der POLAR_MI-Studie eine anspruchsvolle Studienkonzeption entwickelt. Ob das Vorhaben gelingt, hängt von vielen Faktoren ab. Wichtig ist das Zusammenspiel von Stationsapotheker:innen und DIZ vor Ort. Wichtig ist auch die Kooperationsbereitschaft der Universitätskliniken bei der ggf. anstehenden Vorbereitung zur sogenannten Eigenentwicklung nach MDR und dem zugehörigen Qualitätsmanagement. Wir sehen aktuell eine große Bereitschaft, das Vorhaben umzusetzen. Alle Akteur:innen sind sich darüber bewusst, dass die Anforderungen aus INTERPOLAR paradigmatisch für viele andere zukünftige Projekte mit dem Einsatz von IT-Werkzeugen an den Standorten sein werden. INTERPOLAR hat sowohl von den Arbeitsgruppen der Technologie- und Methodenplattform für die vernetzte medizinische Forschung e. V. (TMF) als auch von den MII(Modul 2b)-Beratungsangeboten der Verbundprojekte fit4translation und EVA4MII profitiert.

## Fazit und Ausblick

INTERPOLAR‑1 wird die erste große übergreifende interventionelle Studie in Deutschland sein, mit der überprüft wird, ob Arzneimitteltherapiesicherheit im stationären Bereich durch IT-basierte Aufbereitung von Falldaten verbessert werden kann, indem Stationsapotheker:innen bei der Medikationsanalyse ein IT-Werkzeug zur Verfügung gestellt wird, mit dem sie Patient:innen mit einem vermuteten medikationsbezogenen Problem leichter suchen und identifizieren können. Dieses erleichtert ihnen in Abstimmung mit den verordnenden Ärzt:innen eine Lösung zu finden. Erste Ergebnisse der Studie sind im Jahr 2026 zu erwarten.

Es ist hervorzuheben, dass der Studientyp innovativ ist, da neben dem multizentrischen „stepped-wedge cluster-randomisierten“ Design Daten aus unterschiedlichen Quellen genutzt werden. Daten werden aus dem EMR und aus der Dokumentation der Stationsapotheker:innen fallbezogen in den DIZ zusammengeführt und dann in einer aggregierten Metaanalyse über alle Standorte hinweg ausgewertet. Die Datenqualität wird durch regelmäßige Ausleitungen und Plausibilitätsanalysen sowie Subkohortenanalysen evaluiert.

Über das eigentliche Studienziel von INTERPOLAR‑1 hinaus wird INTERPOLAR Einsichten dazu liefern, ob und wie solche akademisch getriebenen Studien mit Einsatz von IT-Unterstützung in der Routineversorgung und anspruchsvollem Design zukünftig gestaltet werden können und ob der Studientyp zur neuartigen Evidenzbildung sinnstiftend ist.

## Footnotes

1 Kurz für „INTERventional POLypharmacy – Drug InterActions – Risks“.

2 Europäische Screening-Liste zur Identifizierung potenziell unangemessener Arzneimitteltherapien bei älteren Patienten.

3 ATC: anatomisch-therapeutisch-chemisches Klassifikationssystem.

4 LOINC: Logical Observation Identifiers Names and Codes (Datenbank mit allgemeingültigen Namen und Identifikatoren zur Bezeichnung von Untersuchungs- und Testergebnissen aus Labor und Klinik).

5 FHIR: „Fast Healthcare Interoperability Resources“ ist der Standard für ein Austauschformat, der von der US-amerikanischen Organisation HL7 (Health Level Seven International) entwickelt wurde.
